# Evaluation of Knowledge of Patients with Hemophilia Regarding Their Diseases and Treatment in Iran

**DOI:** 10.4274/tjh.2016.0041

**Published:** 2016-12-01

**Authors:** Mehran Karimi, Tahereh Zarei, Sezaneh Haghpanah, Zohreh Zahedi

**Affiliations:** 1 Shiraz University of Medical Sciences, Hematology Research Center, Shiraz, Iran

**Keywords:** Knowledge, Hemophilia, treatment, Disease

## To the Editor,

Hemophilia A and B are hereditary X-chromosomal recessive disorders affecting 1 in 5000 male births [1,2]. Hemophilia is classified as severe at F VIII / F IX <1 kIU L-1, moderate at 1-5 kIU L-1, and mild at >5-25 kIU L-1 [3].

During the mid-1970s hemophilia care underwent substantial improvement to provide more optimal disease management for bleeding prevention strategies and education programs. This led to better educational strategies for disease management [4,5].

Home therapy can be used to manage mild and moderate bleeding episodes and can help to achieve optimal treatment, resulting in decreased pain and hospital admissions for complications [6].

In this cross-sectional study, 30 patients with hemophilia A and B who were registered at the Hemophilia Center of Shiraz, Fars Province, southern Iran, were investigated between March and October of 2013. The data collection form consisted of two parts including demographic data and 22 specific questions regarding assessment of knowledge of the patients regarding the disease and treatment. In this latter section specific topics included appropriate treatment, disease transmission, physiotherapy application, management of bleeding, and the most common symptoms of bleeding.

The correct answer to questions had a sum of 1 to 4 points. Some of the questions had more than one correct answer.

Total knowledge scores were categorized into three grades: scores of 1-14 (poor knowledge), 15-29 (fair knowledge), and 30-41 (good knowledge).

This study was approved by the Ethics Committee of Shiraz University of Medical Sciences.

Data were analyzed by SPSS 17 using the Mann-Whitney U test and the Pearson correlation test, and p<0.05 was considered as statistically significant.

Demographic characteristics of the patients including disease severity and educational level are shown in Table 1.

Participants included 3 female patients and 27 male patients; 26 patients had hemophilia type A and 4 patients had hemophilia type B.

The median age of the patients was 23.5±6.1 years, ranging from 8 to 37 years old. Seven patients had a mild/moderate and 23 had a severe form of hemophilia.

Overall, the mean knowledge score of the patients was determined as 14.7±4.5 (range: 4-26). Considering the three levels of knowledge classification, all patients fell into the first category of poor knowledge (score of <30). There was no significant correlation between the knowledge of the patients and their ages (p=0.094). The results also revealed no significant association between the knowledge of patients and disease severity (p=0.446) or educational level (p>0.999).

There are limited studies that assess the knowledge level of individual patients regarding the management of hemophilia [7,8,9]. An important finding of this study was that patients’ knowledge was not correlated with age, educational level, or disease severity.

Hemophilia associations should be recommended for educational programs for patients and caregivers. Hematologists and nongovernmental organizations can work together for lifelong educational programs. Finally, we recommend holding patient workshops twice a year as well as publishing simple books or brochures in each local language to improve the knowledge and therefore the quality of life of these patients.

## Ethics

Ethics Committee Approval: This study was approved by the Ethics Committee of Shiraz University of Medical Sciences.

## Figures and Tables

**Table 1:**
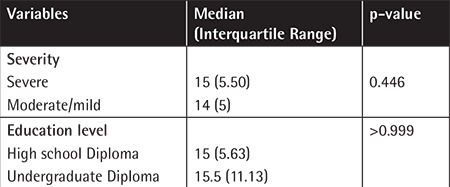
Demographic characteristics of the patients with hemophilia, including severity and educational level.
